# Anticancer effects of 6-shogaol via the AKT signaling pathway in oral squamous cell carcinoma

**DOI:** 10.1590/1678-7757-2021-0209

**Published:** 2021-10-11

**Authors:** Hai HUANG, Myoung-Ok KIM, Ki-Rim KIM

**Affiliations:** 1 Kyungpook National University Department of Animal Science and Biotechnology Sangju Republic of Korea Kyungpook National University, Department of Animal Science and Biotechnology, Sangju, Republic of Korea.; 2 Kyungpook National University Department of Dental Hygiene Sangju Republic of Korea Kyungpook National University, Department of Dental Hygiene, Sangju, Republic of Korea.

**Keywords:** 6-shogaol, Oral squamous cell carcinoma, PI3K/AKT signaling pathway

## Abstract

**Objective:**

Oral squamous cell carcinoma (OSCC) is one of the common type of cancer that leads to death; and is becoming a global concern. Due to the lack of efficient chemotherapeutic agents for patients with oral cancer, the prognosis remains poor. 6-shogaol, a bioactive compound of ginger, has a broad spectrum of bioactivities and has been widely used to relieve many diseases. However, its effects on human oral cancer have not yet been fully evaluated. In our study, we investigated the anticancer effects of 6-shogaol on the proliferation, migration, invasion, apoptosis, and underlying mechanisms within human OSCC cell lines.

**Methodology:**

We investigated the effect of 6-shogaol on the growth of OSCC cells by cell viability and soft agar colony formation assay. Migration and invasion assays were conducted to confirm the effect 6-shogaol on OSCC cell metastasis. Apoptosis was detected by flow cytometry and the underlying mechanism on the antigrowth effect of 6-shogaol in OSCC cells was assessed using western blotting.

**Results:**

In our results, 6-shogaol not only suppressed proliferation and anchorage-independent cell growth in OSCC cells, but also induced apoptosis by regulating the apoptosis-associated factors such as p53, Bax, Bcl-2, and cleaved caspase-3. Migration and invasion of OSCC cells were inhibited following the regulation of E-cadherin and N-cadherin by 6-shogaol. Additionally, 6-shogaol treatment significantly inhibited the PI3K/AKT signaling pathway.

**Conclusion:**

Therefore, our results may provide critical evidence that 6-shogaol can be a potential new therapeutic candidate for oral cancer.

## Introduction

Cancer within the oral cavity and pharynx is one of the most common types worldwide, occurring more often in men than women.^1^ Oral squamous cell carcinoma (OSCC) accounts for more than 90% of all oral cancers and is caused by various factors, including tobacco, alcohol, and heredity.^2^ Since most oral cancers are diagnosed at a late stage, the 5-year survival rate for OSCC patients was reported to be approximately of 55%.^3^ Surgical resection is the primary treatment for most oral cancers, followed by either radiation or chemotherapy, or a combination thereof.^4^ Despite advances in anticancer treatment techniques, patients with oral cancer develop esthetic and functional impairments due to the surgical defect at the head and neck region.^5^ As a result, the patient’s quality of life deteriorates. Therefore, it is important to develop nontoxic and effective new drugs for the prevention and treatment of oral cancer in patients.

Compared to traditional cytotoxic agents, recent oncology has focused on molecules regulating signal transduction pathways, which are key factors in cancer development. Of these pathways, AKT signaling is a central mechanism for carcinogenic activation. The AKT-modified expression has been implicated in various cancers, including esophageal and colon cancer, and is also associated with cell growth, apoptosis or epithelial–mesenchymal transition (EMT) during the process of carcinogenesis.^6,7^ EGFRvIII and phosphorylated AKT are considered as predictive value for patient survival outcome.^8^ Several studies have reported that the phosphatidylinositol 3-kinase (PI3K)/AKT/mammalian target of rapamycin (mTOR) pathway is involved in oral cancer progression and the AKT signaling pathway may be an important therapeutic target of oral cancer.^8-11^

6-shogaol is one of the major physiologically active compounds in dried ginger and has various pharmacological activities, including anti-inflammatory, antioxidant, and antitumor.^12^ In particular, the antitumor activity of 6-shogaol is displayed by regulating signaling molecules such as AKT, mitogen-activated protein kinases, signal transducer and activator of transcription-3 (STAT3), cyclin D1, Bcl-2 and caspases; all of these are associated with cell proliferation, cell cycle arrest, anti-apoptosis, and tumor progression.^11,13,14^ However, few studies have reported on the effect of 6-shogaol in oral cancer.

In our study, we explored the anticancer effects of 6-shogaol on cell proliferation and the mechanisms involved in regulating survival in human OSCC cells.

## Methodology

### Reagents and antibodies

**Figure 1:**
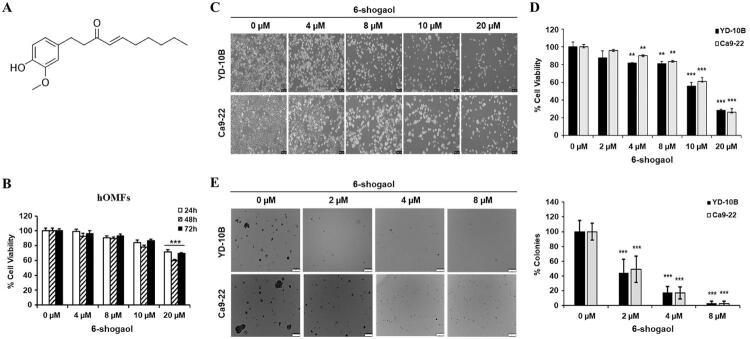
Effects of 6-shogaol on the growth of OSCC cells. (A) The chemical structure of 6-shogaol. (B) The toxicity effect of 6-shogaol on human oral mucosal fibroblasts at different concentrations. (C) The morphological analysis of YD-10B and Ca9-22 after treatment with 6-Shogaol (100×). (D) The viability of YD-10B and Ca9.22 cells treated with various concentrations of 6-shogaol for 24 h using MTT assay. (E) In colony formation assay, representative photographs and quantitative graphs of colonies formed on soft agar from YD-10B and Ca9-22 cells treated with 0, 2, 4, and 8 μM 6-shogaol. Data are expressed as the mean ± SEM. * p<0.05, ** p<0.01, and *** p<0.001 versus control (0 µM 6-shogaol)

6-shogaol (purity ≥ 97% from HPLC and NMR analysis) was obtained from Weikeqi Biological Technology Co. Ltd (Wuhan, Hubei, China) (Figure 1A). Dulbecco’s modified Eagle medium (DMEM), phosphate-buffered saline (PBS), fetal bovine serum (FBS), antibiotic–antimycotic mixture containing 100 U/ml of penicillin and 100 ug/ml of streptomycin, and 0.25% trypsin-ethylenediaminetetraacetic acid (EDTA) were purchased from Gibco BRL. (Grand Island, NY, USA). Anti-p-PI3K, anti-PI3K, anti-p-AKT, anti-AKT, anti-p-mTOR, anti-mTOR, anti-Bax, anti-p53, anti-E-cadherin, and anti-N-cadherin antibodies were obtained from Cell Signaling Technology (Danvers, MA, USA). Anti-actin, anti-Bax, and anti-Bcl-2 antibodies were purchased from Santa Cruz Biotechnology (Santa Cruz, CA, USA). Dimethyl sulfoxide (DMSO) and 3-(4,5-dimethylthiazol-2-yl)-2,5-diphenyltetrazolium bromide (MTT) were purchased from Sigma-Aldrich (St. Louis, MO, USA).

### Cell lines and cell culture

Human OSCC cell lines (YD-10B and Ca9-22) were obtained from the Department of Oral Biology, College of Dentistry, Yonsei University (Seoul, Korea). Human oral mucosal fibroblasts (hOMFs), as a normal oral cells, were purchased from Aiyan Biotech Co., Ltd (Shanghai, China). OSCC cells and hOMFs were cultured in DMEM supplemented with 10% FBS and 1% antibiotic–antimycotic mixture, and incubated in a humidified atmosphere with 5% CO_2_ at 37°C.

### Cell viability assay

Cells (1×10^4^ cells/well) were seeded in 96-well plates and cultured in a complete medium overnight. The attached cells were incubated in serum-free media with varying concentrations of 6-shogaol for 24, 48, and 72 h. MTT solution (5 mg/ml) was added to each well, and incubated at 37°C for 4 h. The medium was subsequently discarded, and DMSO was added to each well to dissolve the formazan crystals. The absorbance was measured at 570 nm using a microplate reader (Thermo Fisher Scientific, Waltham, MA, USA).

### Colony formation assay

OSCC cells (8×10^3^ cells/well), suspended in a complete medium, were added to 0.3% agar with 6-shogaol in the top layer over a base layer of 0.6% agar with 6-shogaol. The cultures were maintained under 5% CO_2_ at 37°C for two weeks, as previously described.^15^ Colonies were subsequently visualized under a microscope and counted using Image-Pro Plus software.

### Cell migration and invasion assay

Transwell chambers (Corning Costar, Lowell, MA, USA) with 8.0 μm pore polycarbonate membrane insert were used for the migration and invasion assay according to the manufacturer’s procedure and published methods.^16^ OSCC cells (1×10^5^ cells/well) were seeded onto the upper surface of 1 mg/ml Matrigel-coated membranes (for invasion assay) or uncoated membranes (for migration assay), and the medium containing 6-shogaol was added to the upper and lower chambers. The non-invaded/migrated cells from the upper surface were removed after 24 h of incubation at 37°C. The invaded/migrated cells on the lower surface were fixed with cold 4% formaldehyde, permeabilized with 100% methanol, and stained with 0.5% crystal violet. The membrane with migrated or invaded cells was imaged using a Leica SP2 confocal microscope (Leica Microsystems, Wetzlar, Germany) and quantified using the Image-Pro Plus software.

### TUNEL assay

Apoptotic cell death was conducted using the terminal deoxynucleotidyl transferase dUTP nick-end labeling (TUNEL) assay. OSCC cells (1×10^5^cells/well) were seeded on chamber slides and incubated in DMEM with 6-shogaol for 24 h. Apoptotic cells were detected with the TUNEL assay using the *in situ* apoptosis detection kit (Takara, Shiga, Japan) according to the manufacturer’s instructions. Cells were stained with DAPI, mounted, and examined using fluorescence microscopy (Leica Microsystems). The percentage of apoptotic cells were estimated using annexin V/propidium iodide (PI) staining as described previously.^17^

### Binding assay

Cell lysates were incubated with 6-shogaol-Sepharose 4B beads (Sepharose 4B only beads served as a negative control) in a lysis buffer containing 50 mM Tris–HCl (pH 7.5), 150 mM NaCl, 5 mM EDTA, 1 mM dithiothreitol, 0.01% NP-40, and 2 mg/ml bovine serum albumin at 4°C rotator overnight. The beads were washed three times with a washing buffer containing 50 mM Tris–HCl (pH 7.5), 150 mM NaCl, 5 mM EDTA, 1 mM dithiothreitol, and 0.01% NP-40. The binding was visualized by western blot analysis with an anti-AKT antibody.

### Western blot analysis

OSCC cells were lysed using a lysis buffer (50 mM Tris–HCl, 150 mM NaCl, 1% NP-40, 1 mM PMSF, protease/phosphatase inhibitor cocktail) for the collection of total protein. The protein concentrations of samples were quantified using the bicinchoninic acid protein assay kit (Thermo Fisher Scientific). Equal amounts of protein were loaded onto a sodium dodecyl sulfate-polyacrylamide gel, electrophoresed, and transferred to a polyvinylidene fluoride membrane (Millipore, Billerica, MA, USA). Membranes were blocked with 5% nonfat dry milk in Tris-buffered saline with 0.1% Tween 20 (TBST) and subsequently incubated with primary antibodies overnight at 4°C. After washing with TBST, the membranes were incubated with HRP-conjugated secondary antibodies for 1 h at room temperature. The detected proteins were visualized using the Amersham enhanced chemiluminescence reagent (GE Healthcare, Little Chalfont, UK) and analyzed using Da Vinci software.

### Statistical analysis

All quantitative results are expressed as the mean ± standard error of three independent experiments. Significant differences were compared using a two-tailed independent sample *t*-test between the different groups, and *p*-values less than 0.05 were considered statistically significant.

## Results

### 6-shogaol inhibits the growth of OSCC cells

To investigate the effect of 6-shogaol on the growth of oral cancer cells, we conducted assays on cell proliferation and colony formation. To first confirm the toxicity of 6-shogaol in normal human oral cells, we measured the viability of hOMFs by treating with different concentrations of 6-shogaol. Figure 1B shows that no toxicity was exhibited in normal oral cells at 0-10 μM 6-shogaol. According to the previously indicated 6-shogaol concentrations, the MTT assay determined the viability of OSCC cells. Figures 1C and 1D show that 6-shogaol dose-dependently suppressed the proliferation of both YD-10B and Ca9-22 cells. A 20 μM concentration of 6-Shogaol inhibited both types of cells by more than 70%. In subsequent experiments, we used the concentration of 6-shogaol with a survival rate of 80% or more. For anchorage-independent cell growth assessment, we conducted the colony formation assay for two weeks; 6-shogaol dose-dependently decreased both the size and the number of colonies in the two cell lines (Figure 1E).

### 6-shogaol suppresses the migration and invasion in OSCC cells

**Figure 2:**
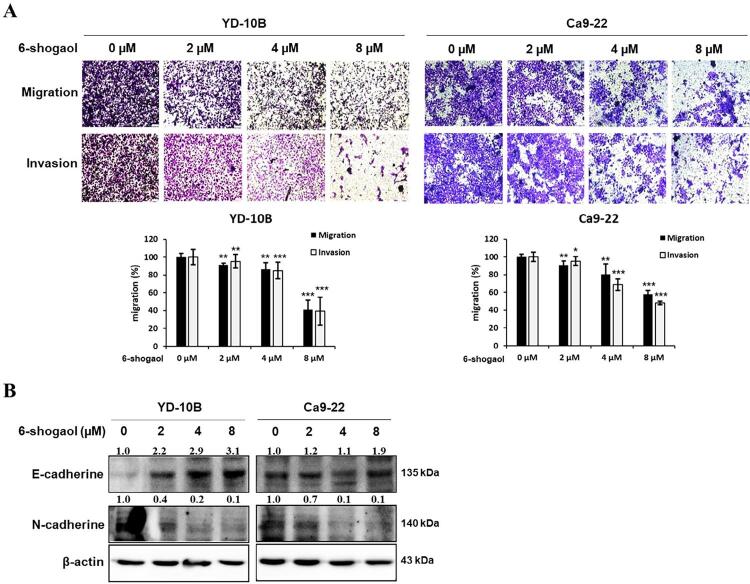
Effects of 6-shogaol on the migration and invasion of OSCC cells. (A) Representative photographs and quantitative graphs of the migrated and invaded YD-10B and Ca9-22 cells treated with 0, 2, 4, and 8 μM 6-shogaol using transwell. Data are expressed as the mean ± SEM. * p<0.05, ** p<0.01, and *** p<0.001 vs control (0 µM 6-shogaol). (B) Western blot analysis of E-cadherin and N-cadherin expression by 6-shogaol treatment in YD-10B and Ca9-22 cells

To confirm the effects of 6-shogaol on oral cancer cell metastasis, we investigated the migration and invasive abilities of OSCC cells and the expression EMT-related proteins, which play an essential role in local recurrence and lymph node metastasis of cancer. The results indicated that the ability of OSCC cells to migrate and invade was dose-dependently reduced by 6-shogaol as compared to the control (0 μM of 6-shogaol) (Figure 2A). Quantitative analysis showed that the migration and invasion were reduced by approximately 60%, using 8 μM concentration of 6-shogaol in both OSCC cell lines. Treatment with 6-shogaol dose-dependently induced an increase in E-cadherin and a decrease in N-cadherin. These results inhibited the migration and invasion of OSCC cells (Figure 2B). Thus, 6-shogaol may suppress the EMT process of human OSCC cells.

### 6-shogaol induces the apoptosis in OSCC cells

**Figure 3:**
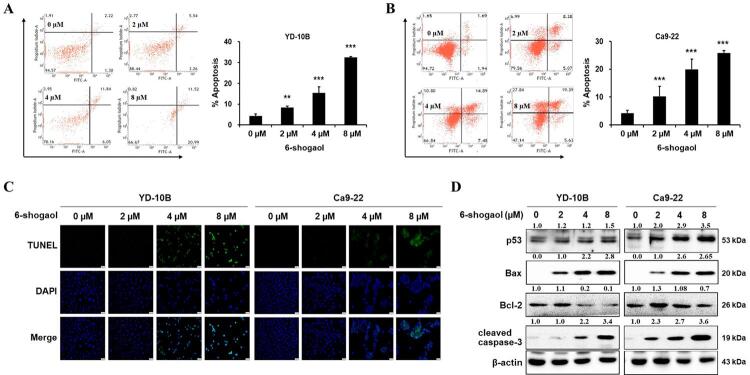
Effects of 6-shogaol on the apoptosis of OSCC cells. (A and B) Annexin V staining and quantification of apoptosis rates in YD-10B and Ca9-22 cells by 6-shogaol treatment. Data are expressed as the mean ± SEM. * p<0.05, ** p<0.01, and *** p<0.001 vs control (0 µM 6-shogaol). (C) The apoptosis phenomenon of YD-10B and Ca9-22 cells treated with 6-shogaol using the TUNEL assay. (D) The expression of cell apoptosis markers, including p53, Bax, Bcl-2, and cleaved caspase-3 in 6-shogaol-treated OSCC cells by western blotting

To investigate whether apoptosis affected the antigrowth effect of 6-shogaol, we conducted Annexin V/PI detection and TUNEL assay in two OSCC cell lines. 6-shogaol-treated OSCC cells were double-stained with annexin V-FITC and PI, and analyzed using a flow cytometry. 6-shogaol remarkably induced both early and late stages of apoptosis in OSCC cells in a dose-dependent manner compared to the control (Figure 3A–B). The apoptotic populations treated by concentrations of 2, 4, and 8 µM of 6-shogaol were measured to be approximately 10%, 20%, and 30% in both YD-10B and Ca9-22 cells. From the results of the TUNEL assay, it was clear that treatment with 6-shogaol dose-dependently raised the apoptosis rate of OSCC cells compared to the control group (Figure 3C). To confirm the mechanism of apoptosis which was induced by 6-shogaol, the level of apoptosis-related proteins was also investigated using western blotting. 6-shogaol treatment significantly increased the expression of p53, Bax, and cleaved caspase-3 in a dose-dependent manner, but decreased the anti-apoptotic protein Bcl-2 (Figure 3C). These results suggest that 6-shogaol significantly induces the apoptosis of OSCC cells.

### 6-shogaol blocks the PI3K/AKT signaling pathway in OSCC cells

**Figure 4:**
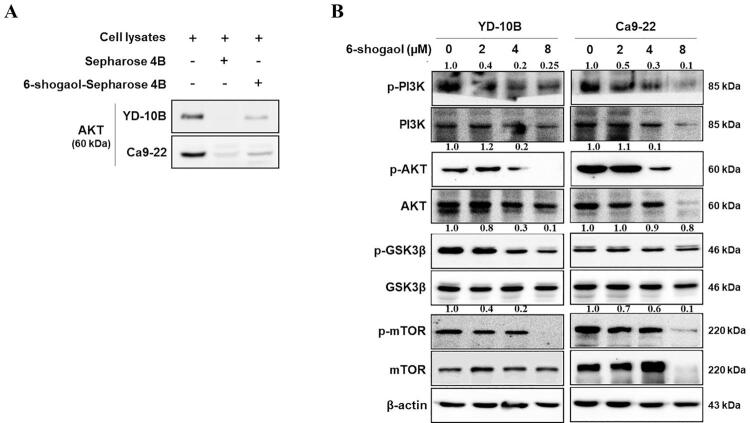
Effects of 6-shogaol on the PI3K/AKT signaling pathway in OSCC cells. (A) Pull-down assay showing 6-shogaol bound to AKT. (B) Western blot analysis of the expressions of proteins related to the PI3K/AKT/mTOR signaling pathway in OSCC cells treated by 6-shogaol

To elucidate the regulatory mechanism underlying the antigrowth effect of 6-shogaol in OSCC cells, we assessed the expression of critical PI3K/AKT pathway proteins. We conducted an *ex vivo* pull-down assay with OSCC cell lysates to establish whether AKT is a target of 6-shogaol. We identified that 6-shogaol did indeed bind with AKT directly (Figure 4A). Significant proteins involved in the PI3K/AKT/mTOR signaling pathway were subsequently analyzed using western blotting. Figure 4B shows that 6-shogaol significantly decreased the expression of p-PI3K, p-AKT, and p-mTOR in a dose-dependent manner in both YD-10B and Ca9-22 cells. However, no significant change in the total protein expression of PI3K, AKT, and mTOR was observed. Additionally, glycogen synthase kinase 3beta (GSK3β) was also inhibited following 6-shogaol treatment. These results indicate that 6-shogaol may increase apoptosis of OSCC cells through the PI3K/AKT/mTOR signaling pathway.

## Discussion

Oral cancer is a common fatal cancer and remains a major threat to global public health. Local recurrence and lymph node metastasis are considered the mainly responsible for the low survival rate in patients with OSCC.^18,19^ Therefore, although current therapeutic methods, such as surgical resection and radiotherapy, are necessary, it is difficult to obtain satisfactory results because of local recurrence and metastasis of cervical lymph nodes after surgery.^20^ Furthermore, despite the advances in drugs for various cancers, adjuvant chemotherapy for oral cancer patients has not yet been established.^21^ Recently, compounds isolated from plants or fruits have garnered interest due to their comparatively lower toxic effects and multiple targets.^22^ Therefore, many researchers are investigating natural products to develop effective prevention and therapeutic agents for various cancers. However, there are few studies on the efficacy of natural products for oral cancer. Here, we introduced 6-shogaol, one of the main compounds in dried ginger, which was reported to possess activities in cancer prevention.^13,23,24^ The anticancer ability of 6-shogaol has been studied in various cancers;^24-26^ however, the mechanism of 6-shogaol in oral cancer has not yet been reported.

Previous reports showed that 6-shogaol plays a crucial role in kidney cancer osteoclastogenic activity and metastatic potential by suppressing 2-Amino-1-methyl-6-phenylimidazo [4,5-b]pyridine (PhIP).^27^ Bawadood et al. reported that 6-shogaol induces apoptosis by targeting notch signaling pathway, which therefore suppresses breast cancer cells proliferation and autophagy.^28^ It has been shown that targeting the TLR4 signaling pathway with 6-shogaol plays an important role in cancer therapy.^29^ Back to our concern, our findings suggest that 6-shogaol may affect oral cancer cell growth by inhibiting AKT pathway. Notably, 6-shogaol is a multi-targeted drug.

Our study showed that 6-shogaol inhibits cell proliferation by inducing apoptotic cell death in two different human OSCC cell lines, YD-10B and Ca9-22. The regulation of apoptotic gene expression, such as the increase in p53, cleaved caspase-3, and Bax along with the decrease in Bcl-2, supports the antitumor effect of 6-shogaol on OSCC cells. Moreover, the EMT event is a pivotal process in tumorigenesis and metastasis.^30,31^ To further understand EMT mechanisms in OSCC cells, we conducted a migration and invasion assay using transwell with and without Matrigel in YD-10B and Ca9-22 cells and confirmed the expression of EMT-related proteins. In the two OSCC cell lines, 6-shogaol treatment significantly suppressed the ability of migration and invasion of the cells; resulting in an alteration in the expression of EMT markers, including promotion of E-cadherin expression and inhibition of N-cadherin expression compared to the control group. These results indicate the inhibitory effect of 6-shogaol on the EMT process in oral cancer and also suggest a potential therapeutic value of 6-shogaol for treating oral cancer.

The mechanism of action assays for 6-shogaol in various cancer cells showed that it is involved in multiple signaling pathways during initiation, progression, and metastasis.^26-29,32^ As one of the important pathways in tumorigenesis, once the PI٣K/AKT/mTOR signaling pathway is activated, various oncogenes that can lead to cancer progression are upregulated.^33^ Yang and Wang investigated the role of AKT in oral cancer using different compounds.^9,34^ Kim, et al.^11^ (2014) reported that 6-shogaol could inhibit cell proliferation in lung cancer by directly targeting AKT. Studies have also reported that 6-shogaol reduces the survival of fibrosarcoma cells by inducing an increase in ROS production and activation of AKT/mTOR.^35^ Furthermore, Matsuo, et al.^36^ (2018) reported the pathologic significance of AKT, mTOR, and GSK3β proteins in OSCC patients, and confirmed that GSK3β drove the cervical lymph node metastatic spread of OSCC cells. Therefore, AKT, mTOR and GSK3β at the process of carcinogenesis were appreciated as target molecules for the prevention and treatment of oral cancer.^37^

**Figure 5:**
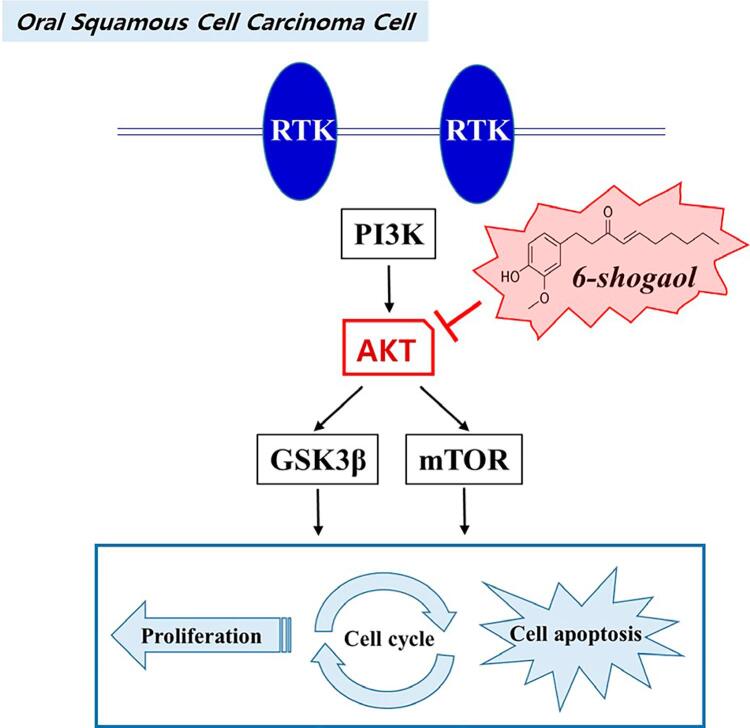
A proposed schematic diagram illustrating the roles of AKT signaling pathway by 6-shogaol in OSCC cells. 6-shogaol exerts the anticancer effects by regulating cell proliferation, cell cycle arrest, and apoptosis via PI3K/AKT signaling pathway by targeting AKT

In our study, we confirmed that 6-shogaol could directly bind with AKT in oral cancer cells and inhibit the downstream effect of AKT. 6-shogaol significantly reduced the expressions of p-PI3K, p-AKT, and p-mTOR in OSCC cells; and also inhibited GSK3β downstream of AKT, which plays an important role in the proliferation and apoptosis of cancer cells. As in the aforementioned anticancer studies of 6-shogaol in various cancer cells, in our study, 6-shogaol also inhibited the PI3K/AKT pathway, thus inhibiting cell proliferation and inducing apoptosis (Figure 5). Undoubtedly, targeting AKT with 6-shogaol will help treat oral cancer. Therefore, AKT could be a novel biomarker for diagnosis and prognosis and could serve as a therapeutic target for patients with oral cancer. Taken together, 6-shogaol inhibited cell proliferation, migration, and invasion as well as promoted the apoptosis of OSCC cells. These results indicate that 6-shogaol may be a potential new therapeutic agent for patients with oral cancer.

## Conclusion

Our study shows that 6-shogaol suppresses the proliferation of two OSCC cell lines, YD-10B and Ca9-22, in a dose-dependent manner by targeting AKT. The results also indicate that 6-shogaol inhibits the EMT process and triggered apoptosis by activating the PI3K/AKT/mTOR pathway in OSCC cells. Our findings support the development of natural product-derived compound as a new therapeutic candidate for patients with oral cancer. However, since these are cellular-level results, *in vivo* studies should be further evaluated for the potential application of 6-shogaol in the prevention and treatment of oral cancer.

## References

[1] 1 - Siegel RL, Miller KD, Jemal A. Cancer statistics, 2020. CA Cancer J Clin. 2020;70(1):7-30. doi: 10.3322/caac.21590 10.3322/caac.21590 31912902

[2] 2 - Ettinger KS, Ganry L, Fernandes RP. Oral cavity cancer. Oral Maxillofac Surg Clin North Am. 2019;31(1):13-29. doi: 10.1016/j.coms.2018.08.002 10.1016/j.coms.2018.08.002 30454788

[3] 3 - Güneri P, Epstein JB. Late stage diagnosis of oral cancer: components and possible solutions. Oral Oncology. 2014;50(12):1131-6. doi: 10.1016/j.oraloncology.2014.09.005 10.1016/j.oraloncology.2014.09.005 25255960

[4] 4 - Nandini DB, Rao RS, Hosmani J, Khan S, Patil S, Awan KH. Novel therapies in the management of oral cancer: an update. Dis Mon. 2020;66(12):101036. doi: 10.1016/j.disamonth.2020.101036 10.1016/j.disamonth.2020.101036 32594997

[5] 5 - Hassanein KA, Musgrove BT, Bradbury E. Functional status of patients with oral cancer and its relation to style of coping, social support and psychological status. Br J Oral Maxillofac Surg. 2001;39(5):340-5. doi: 10.1054/bjom.2001.0652 10.1054/bjom.2001.0652 11601811

[6] 6 - Song M, Liu X, Liu K, Zhao R, Huang H, Shi Y, et al. Targeting AKT with Oridonin inhibits growth of esophageal squamous cell carcinoma *in vitro* and patient-derived xenografts *in vivo*. Mol Cancer Ther. 2018;17(7):1540-53. doi: 10.1158/1535-7163.Mct-17-0823 10.1158/1535-7163.MCT-17-0823 PMC6715294 29695636

[7] 7 - Suman S, Das TP, Sirimulla S, Alatassi H, Ankem MK, Damodaran C. Withaferin-A suppress AKT induced tumor growth in colorectal cancer cells. Oncotarget. 2016;7(12):13854-64. doi: 10.18632/oncotarget.7351 10.18632/oncotarget.7351 PMC4924683 26883103

[8] 8 - Chang KY, Tsai SY, Chen SH, Tsou HH, Yen CJ, Liu KJ, et al. Dissecting the EGFR-PI3K-AKT pathway in oral cancer highlights the role of the EGFR variant III and its clinical relevance. J Biomed Sci. 2013;20(1):43. doi: 10.1186/1423-0127-20-43 10.1186/1423-0127-20-43 PMC3710269 23806066

[9] 9 - Wang P, Gao WY, Wang YB, Wang J. Acetylshikonin inhibits *in vitro* and *in vi*vo tumorigenesis in cisplatin-resistant oral cancer cells by inducing autophagy, programmed cell death and targeting m-TOR/PI3K/Akt signalling pathway. J BUON. 2019;24(5):2062-7. 31786876

[10] 10 - Wang R, Lu X, Yu R. Lycopene inhibits epithelial-mesenchymal transition and promotes apoptosis in oral cancer via PI3K/AKT/m-TOR signal pathway. Drug Des Devel Ther. 2020;14:2461-71. doi: 10.2147/DDDT.S251614 10.2147/DDDT.S251614 PMC7321693 32606612

[11] 11 - Kim MO, Lee MH, Oi N, Kim SH, Bae KB, Huang Z, et al. [6]-Shogaol inhibits growth and induces apoptosis of non-small cell lung cancer cells by directly regulating Akt1/2. Carcinogenesis. 2014;35(3):683-91. doi: 10.1093/carcin/bgt365 10.1093/carcin/bgt365 PMC3941745 24282290

[12] 12 - Kou X, Wang X, Ji R, Liu L, Qiao Y, Lou Z, et al. Occurrence, biological activity and metabolism of 6-shogaol. Food Function. 2018;9(3):1310-27. doi: 10.1039/c7fo01354j 10.1039/c7fo01354j 29417118

[13] 13 - Li TY, Chiang BH. 6-shogaol induces autophagic cell death then triggered apoptosis in colorectal adenocarcinoma HT-29 cells. Biomed Pharmacother. 2017;93:208-17. doi: 10.1016/j.biopha.2017.06.038 10.1016/j.biopha.2017.06.038 28641163

[14] 14 - Kim SO, Kim MR. [6]-Gingerol prevents disassembly of cell junctions and activities of MMPs in invasive human pancreas cancer cells through ERK/NF-κB/Snail signal transduction pathway. Evid Based Complement Alternat Med. 2013;2013:761852. doi: 10.1155/2013/761852 10.1155/2013/761852 PMC3800596 24204396

[15] 15 - Zhang H, Yi J, Kim E, Choo Y, Hai H, Kim K, et al. 20(S)-Ginsenoside Rh2 suppresses oral cancer cell growth by inhibiting the Src-Raf-ERK signaling pathway. Anticancer Res. 2021;41(1):227-35. doi: 10.21873/anticanres.14769 10.21873/anticanres.14769 33419817

[16] 16 - Chen L, Bi SN, Hou JZ, Zhao ZJ, Wang CJ, Xie SQ. Targeting p21-activated kinase 1 inhibits growth and metastasis via Raf1/MEK1/ERK signaling in esophageal squamous cell carcinoma cells. Cell Commun Signal. 2019;17(1):31. doi: 10.1186/s12964-019-0343-5 10.1186/s12964-019-0343-5 PMC6458688 30971268

[17] 17 - Yin S, Song M, Zhao R, Liu X, Kang WK, Lee JM, et al. Xanthohumol inhibits the growth of keratin 18-overexpressed esophageal squamous cell carcinoma *in vitro* and *in vivo*. Front Cell Dev Biol. 2020;8:366. doi: 10.3389/fcell.2020.00366 10.3389/fcell.2020.00366 PMC7248302 32509787

[18] 18 - Liang J, Liang L, Ouyang K, Li Z, Yi X. MALAT1 induces tongue cancer cells’ EMT and inhibits apoptosis through Wnt/β-catenin signaling pathway. J Oral Pathol Med. 2017;46(2):98-105. doi: 10.1111/jop.12466 10.1111/jop.12466 27353727

[19] 19 - Attramadal CG, Kumar S, Boysen ME, Dhakal HP, Nesland JM, Bryne M. Tumor budding, EMT and cancer stem cells in T1-2/N0 oral squamous cell carcinomas. Anticancer Res. 2015;35(11):6111-20. 26504037

[20] 20 - Noguti J, Moura CF, Jesus GP, Silva VH, Hossaka TA, Oshima CT, et al. Metastasis from oral cancer: an overview. Cancer Genomics Proteomics. 2012;9(5):329-35. 22990112

[21] 21 - Hartner L. Chemotherapy for oral cancer. Dent Clin North Am. 2018;62(1):87-97. doi: 10.1016/j.cden.2017.08.006 10.1016/j.cden.2017.08.006 29126496

[22] 22 - Surh YJ. Cancer chemoprevention with dietary phytochemicals. Nat Rev Cancer. 2003;3(10):768-80. doi: 10.1038/nrc1189 10.1038/nrc1189 14570043

[23] 23 - Yang LL, Yang F, Teng LT, Katayama I. 6-Shogaol protects human melanocytes against oxidative stress through activation of the Nrf2-antioxidant response element signaling pathway. Int J Mol Sci. 2020;21(10):3537. doi: 10.3390/ijms21103537 10.3390/ijms21103537 PMC7279012 32429495

[24] 24 - Liang T, He Y, Chang YH, Liu XT. 6-shogaol a active component from ginger inhibits cell proliferation and induces apoptosis through Inhibition of STAT-3 translocation in ovarian cancer cell lines (A2780). Biotechnol Bioprocess Eng. 2019;24(3):560-7. doi: 10.1007/s12257-018-0502-3

[25] 25 - Chen CY, Yang YH, Kuo SY. Effect of [6]-Shogaol on cytosolic Ca2+ levels and proliferation in human oral cancer cells (OC2). J Nat Prod. 2010;73(8):1370-4. doi: 10.1021/np100213a 10.1021/np100213a 20669930

[26] 26 - Tan BS, Kang O, Mai CW, Tiong KH, Khoo AS, Pichika MR, et al. 6-Shogaol inhibits breast and colon cancer cell proliferation through activation of peroxisomal proliferator activated receptor γ (PPARγ). Cancer Lett. 2013;336(1):127-39. doi: 10.1016/j.canlet.2013.04.014 10.1016/j.canlet.2013.04.014 23612072

[27] 27 - Yeh IJ, Chen SC, Yen MC, Wu YH, Hung CH, Kuo PL. 6-Shogaol suppresses 2-Amino-1-Methyl-6-Phenylimidazo [4,5-b] Pyridine (PhIP)-Induced human 786-O renal cell carcinoma osteoclastogenic activity and metastatic potential. Nutrients. 2019;11(10):2306. doi: 10.3390/nu11102306 10.3390/nu11102306 PMC6835604 31569368

[28] 28 - Bawadood AS, Al-Abbasi FA, Anwar F, El-Halawany AM, Al-Abd AM. 6-Shogaol suppresses the growth of breast cancer cells by inducing apoptosis and suppressing autophagy via targeting notch signaling pathway. Biomed Pharmacother. 2020;128:110302. doi: 10.1016/j.biopha.2020.110302 10.1016/j.biopha.2020.110302 32505819

[29] 29 - Chen CY, Kao CL, Liu CM. The cancer prevention, anti-inflammatory and anti-oxidation of bioactive phytochemicals targeting the TLR4 signaling pathway. Int J Mol Sci. 2018;19(9):2729. doi: 10.3390/ijms19092729 10.3390/ijms19092729 PMC6164406 30213077

[30] 30 - De Craene B, Berx G. Regulatory networks defining EMT during cancer initiation and progression. Nat Rev Cancer. 2013;13(2):97-110. doi: 10.1038/nrc3447 10.1038/nrc3447 23344542

[31] 31 - Brabletz T, Kalluri R, Nieto MA, Weinberg RA. EMT in cancer. Nat Rev Cancer. 2018;18(2):128-34. doi: 10.1038/nrc.2017.118 10.1038/nrc.2017.118 29326430

[32] 32 - Nazim UM, Park SY. Attenuation of autophagy flux by 6-shogaol sensitizes human liver cancer cells to TRAIL-induced apoptosis via p53 and ROS. Int J Mol Med. 2019;43(2):701-8. doi: 10.3892/ijmm.2018.3994 10.3892/ijmm.2018.3994 PMC6317668 30483736

[33] 33 - Shaw RJ, Cantley LC. Ras, PI(3)K and mTOR signalling controls tumour cell growth. Nature. 2006;441(7092):424-30. doi: 10.1038/nature04869 10.1038/nature04869 16724053

[34] 34 - Yang J, Ren XY, Zhang LP, Li YY, Cheng B, Xia J. Oridonin inhibits oral cancer growth and PI3K/Akt signaling pathway. Biomed Pharmacother. 2018;100:226-32. doi: 10.1016/j.biopha.2018.02.011 10.1016/j.biopha.2018.02.011 29432993

[35] 35 - Romero-Arias AC, Sequeda-Castañeda LG, Aristizábal-Pachón AF, Morales L. Effect of 6-shogaol on the glucose uptake and survival of HT1080 fibrosarcoma cells. Pharmaceuticals. 2019;12(3):131. doi: 10.3390/ph12030131 10.3390/ph12030131 PMC6789756 31505728

[36] 36 - Matsuo FS, Andrade MF, Loyola AM, da Silva SJ, Silva MJB, Cardoso SV, et al. Pathologic significance of AKT, mTOR, and GSK3β proteins in oral squamous cell carcinoma-affected patients. Virchows Arch. 2018;472(6):983-97. doi: 10.1007/s00428-018-2318-0 10.1007/s00428-018-2318-0 29713826

[37] 37 - Harsha C, Banik K, Ang HL, Girisa S, Vikkurthi R, Parama D, et al. Targeting AKT/mTOR in oral cancer: mechanisms and advances in clinical trials. Int J Mol Sci. 2020;21(9):3285. doi: 10.3390/ijms21093285 10.3390/ijms21093285 PMC7246494 32384682

